# ELAVL1 Role in Cell Fusion and Tunneling Membrane Nanotube Formations with Implication to Treat Glioma Heterogeneity

**DOI:** 10.3390/cancers12103069

**Published:** 2020-10-21

**Authors:** Natalia Filippova, Louis B. Nabors

**Affiliations:** Department of Neurology, School of Medicine, University of Alabama at Birmingham, Birmingham, AL 35294, USA

**Keywords:** cell-to-cell communication, tumor microenvironment, glioma, HuR, heterogeneity, tunneling membrane nanotubes, cell fusion, inhibitors

## Abstract

**Simple Summary:**

Despite the numerous novel pharmacological and immunological approaches for multimodal glioma treatments that have been proposed in recent years, glioma phenotypic and genotypic spatial profiles in the course of treatments remain heterogeneous and, therefore, represent the biggest challenge for patient outcome. The elimination of glioma heterogeneity is an established chemotherapeutic goal. The role of the mRNA-binding protein of ELAV-family HuR in homotypic and heterotypic cell fusions via permanent intercellular membrane fusions and temporal intercellular tunneling nanotube formations in the glioma microenvironment leading to glioma heterogeneity will be discussed in our review with implications of HuR inhibitors in the prevention of these processes.

**Abstract:**

Homotypic and heterotypic cell fusions via permanent membrane fusions and temporal tunneling nanotube formations in the glioma microenvironment were recently documented in vitro and in vivo and mediate glioma survival, plasticity, and recurrence. Chronic inflammation, a hypoxic environment, aberrant mitochondrial function, and ER stress due to unfolded protein accumulation upregulate cell fusion events, which leads to tumor heterogeneity and represents an adaptive mechanism to promote tumor cell survival and plasticity in cytotoxic, nutrient-deprived, mechanically stressed, and inflammatory microenvironments. Cell fusion is a multistep process, which consists of the activation of the cellular stress response, autophagy formation, rearrangement of cytoskeletal architecture in the areas of cell-to-cell contacts, and the expression of proinflammatory cytokines and fusogenic proteins. The mRNA-binding protein of ELAV-family HuR is a critical node, which orchestrates the stress response, autophagy formation, cytoskeletal architecture, and the expression of proinflammatory cytokines and fusogenic proteins. HuR is overexpressed in gliomas and is associated with poor prognosis and treatment resistance. Our review provides a link between the HuR role in the regulation of cell fusion and tunneling nanotube formations in the glioma microenvironment and the potential suppression of these processes by different classes of HuR inhibitors.

## 1. Introduction

Glioma is the most devastating and incurable disease with a relative risk of 3–4 timers higher in the elderly population than young adults. It is characterized by high tissue heterogeneity and undergoes a fast transformation from low-grade (I–II) to high-grade (III–IV) malignancy [1,2,3]. The transition to a high-grade tumor occurs in approximately 95% of low-grade gliomas; less than 3% of these patients remain alive after five years post-diagnosis [3,4].

Comprehensive genomic profiling of low- and high-grade gliomas confirmed an increase in glioma heterogeneity with tumor grade and treatment resistance. Glioma heterogeneity represents the biggest challenge for glioma treatment, which depends on tumor grade and is customized to individual patients. Surgery, radiotherapy, and chemotherapy with temozolomide (TMZ) represent the first line of treatments for glioma [5,6]. More modern treatments consist of treatment with the genetically engineered autologous or allogeneic T cells expressing chimeric antigen receptors (CARs) directed against specific antigens, the combinations of Optune treatment (an application of the electric field, which induces apoptosis in rapidly dividing tumor cells) and TMZ, the combination of the ketogenic diet with glutamine antagonist 6-diazo-5-oxo-l-norleucine, which induces energy depletion and destroys the mesenchymal type of glioma stem cells [7,8,9]. However, due to tumor heterogeneity, genomic instability, and transforming ability, the current tumor treatments are efficient only in terms of the prolonging time of relapse; therefore, gliomas remain incurable [1,2,3,5].

The needs in new glioma treatment modalities, which eliminate the development of tumor heterogeneity and transforming ability, are evident. Intercellular gene transfers between glioma and normal host cells in the glioma microenvironment via permanent cell fusion and temporal tunneling tube formations have been recently discovered and may lead to a de novo glioma genotype and phenotype inside of the progressing tumor. Our review overviews mechanisms and cell-signaling pathways, which lead to cell fusion and tunneling membrane tube formations in the glioma microenvironment and discusses pharmacological approaches of the interception of these processes.

## 2. Historical Background

Cell fusions via permanent intercellular membrane fusions were discovered more than a century ago under normal physiological conditions such as tissue and organ development, cell fertilization, tissue repair and regeneration, and angiogenesis [10,11]. Only select types of mammalian cells (to include bone marrow-derived mesenchymal stem cells, bone marrow-derived endothelial progenitor cells recruited to the new blood vessels, epithelial cells of the placenta, fiber cells in the eye, macrophages at sites of chronic inflammation, skeletal muscle cells, and gametes) undergo permanent intercellular membrane fusion under normal physiological conditions [12]. The temporal cell fusions via tunneling membrane nanotube formations between mammalian cells were first discovered by Rustom and colleagues in 2004 [13] and occur between different cell types (to include monocytes, lymphocytes, neurons, astrocytes, cardiomyocytes, endothelial cells, fibroblasts, and mesenchymal stem cells under diverse stress conditions) [13,14,15,16,17,18,19]. Tunneling membrane nanotubes vary in length from 5 to 100 μm, and in width from 50 nm to up to 1 μm. Depending on the interconnected cell types and the tunneling nanotube compositions and sizes, the following terms have been utilized to reference the tunneling membrane tube structures: tunneling membrane nanotubes, cytoplasmic intercellular bridges, intercellular nanotubular highways, membrane microtubes [20]. The tunneling membrane nanotubes are not adherent to the substrate and have a lifetime from a couple of minutes to several hours. Under normal physiological conditions, bidirectional intercellular transfers of cellular materials via permanent cell fusion and temporal tunneling nanotube formations lead to tissue development and repair [17,18,19]. However, under pathological conditions such as viral infection, chronic inflammation, diabetes, and cancer, the intercellular transfers of biological materials (including chromosomal and extrachromosomal genetic materials, cellular organelles, viruses) via permanent and temporal cell fusions lead to de novo cell transformations and tissue heterogeneity, and thus to the development of drug resistance and disease progression [20,21,22,23,24,25,26].

## 3. Expression of Fusogens, Fusogen Receptors, and Tunneling Membrane Nanotube-Related Transcripts in Gliomas

**Figure 1:**
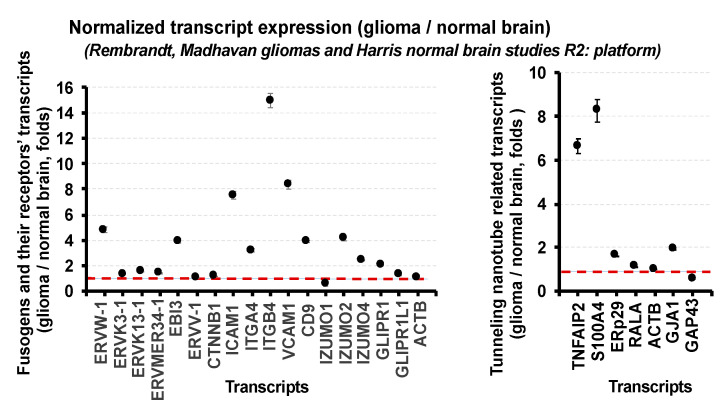
Expression of fusogens and the tunneling membrane nanotube-related transcripts in gliomas compared to normal brain. Graphs illustrate normalized expression of the fusogens and their receptors’ transcripts (**left**) and the tunneling membrane nanotube-related transcripts (**right**) in gliomas compared to normal brain. The following studies from the R2: platform have been utilized for the analysis: Rembrandt, Madhavan, Mas 5.0-U132p2 study for gliomas and Harris, Mas 5.0-U132p2 study for normal brain. Data are shown as mean ± SD. Detailed methods associated with transcriptomic profiling were provided in the R: platform (Rembrandt, Madhavan, gliomas and Harris, normal brain studies).

Both intercellular tunneling nanotubes and permanent intercellular membrane fusions are reported in gliomas and represent diverse multistep processes, which require an activation of the cellular stress response, the rearrangement of the actin-dependent cytoskeleton, the expression of the fusogenic proteins, and phosphatidylserine enrichment on the membrane surfaces [24,25,26,27,28,29,30,31,32,33,34,35]. We utilized the literature search and the R2: Genomics Analysis and Visualization Platform (Rembrandt, Madhavan, Mas 5.0-U132p2 study for gliomas and Harris, Mas 5.0-U132p2 study for normal brain) and confirmed the significant expression of the following known fusogens in the glioma microenvironment: (i) the fusogen transcripts from genomes of pathogenic HCMV, HHV-6, HIV1, and Epstein–Barr enveloped viruses; (ii) the fusogen transcripts encoding endogenous retroviral envelope proteins (ERVW-1, ERVK13-1, ERV3-1, ERVMER34-1, ERVV-1, ERVFRD-1); (iii) the fusogen transcripts encoding proteins essential for sexual reproduction and gamete fusions (transcripts of IZUMO and IZUMOR families, GLIPR1L1, CD9); (iv) the muscle-specific fusogen transcripts (myomaker and myomixer) at low levels; (v) the transcripts of fusogens involved in intercellular and extracellular vesicle-specific transfers (SNARE-family transcripts, transcripts of small dynamin-like GTPases including atlastins, mitofusins, dynamins). The type 1 tunneling membrane nanotube-related transcripts (TNFAIP2, S100A4, ERp29) [36,37,38,39] were significantly overexpressed in gliomas compared to normal brain. The type 2 tunneling nanotube transcripts (GJA1, GAP43) [40,41], which overlap with gap junction formations, exhibited a diverse expression in gliomas (GJA1 transcript was significantly overexpressed; however, GAP43 expression was significantly decreased compared to normal brain samples). It will be important to mention that the higher tumor grades correlated with the significant increase in the TNFAIP2 and S100A4 transcript expression. The Ttyh1 transcript, a biomarker of the recently identified the Ttyh1-dependent subset of the tunneling microtubes with widths up to 2.5 μm [32,42], exhibited significant overexpression in gliomas compared to normal brain. However, it was reported that Ttyh1 is downregulated in 1p/19q-co-deleted versus 1p/19q intact human gliomas by utilizing RNA-Seq gene-expression analysis [32]. Because of deficient Thyh1-dependent tunneling microtube formation and function, 1p/19q-co-deleted oligodendrogliomas have been reported as invasion-deficient [43], suggesting the diverse roles of tunneling nanotube and tunneling microtubes in gliomas with different genetic backgrounds [32,43]. Figure 1 illustrates the expression of fusogens, fusogen receptors, and the tunneling membrane nanotube type 1- and type 2-related transcripts in gliomas normalized to the expression of the corresponding transcripts in the normal brain and the corresponding expression of the house-keeping gene ACTB (actin).

The publicly available transcriptomic data (RNA-seq) of five patient-derived glioma xenograft (PDGx) neurosphere cell lines of classic, proneural, and mesenchymal subtypes presented in the Gene Expression Omnibus (GEO) repository database (GSE158271) were utilized for the analysis of the expression of the fusogens and the tunneling nanotube-related transcripts in gliomas of different subtypes. The analyses of the transcriptome signatures of five PDGx neurosphere cell lines confirmed the expression of a variety of fusogen and tunneling membrane nanotube-related transcripts in all glioma subtypes. Table 1 and Table 2 provide a summary of the normalized to the house-keeping gene ACTB transcript expression in the different glioma subtypes.

Detailed methods associated with transcriptomic profiling were provided in the Gene Expression Omnibus repository database (GSE158271). The expression levels were defined as (i) strong (+++) if the expression was in the 70–100% range of the maximum expression of the corresponding transcripts; (ii) medium (++) if the expression was in the 30–70% range of the maximum expression of the corresponding transcripts; (iii) weak (+) if the expression was in the 1–30% range of the maximum expression of corresponding transcripts.

**Figure 2:**
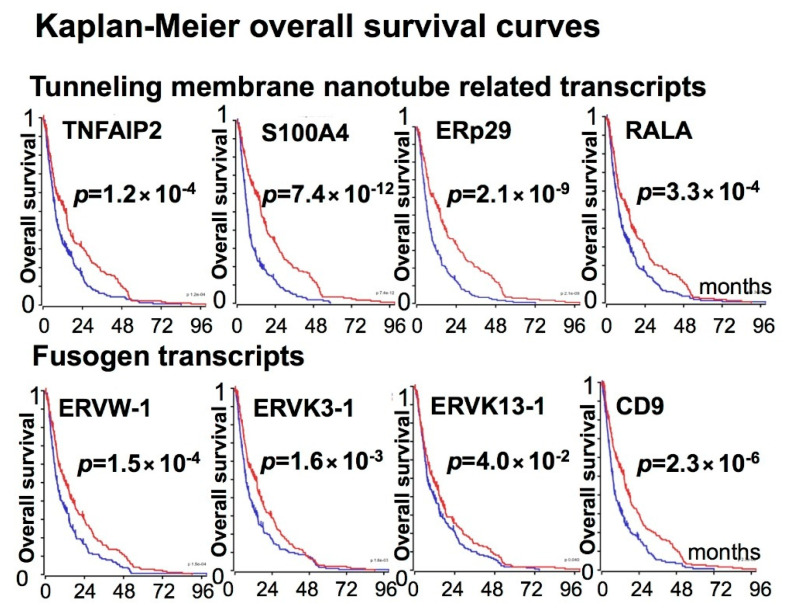
Expression of the tunneling membrane nanotube-related transcripts (TNFAIP2, S100A4, ERp29, RALA) and the fusogen transcripts (ERVW-1, ERVK3-1, ERVK13-1, CD9) significantly decreased overall survival of glioma patients. Graphs illustrate the Kaplan–Meier overall survival curves for patients with gliomas harboring low (red) or high (blue) expression of the corresponding transcripts (Rembrandt, Madhavan, Mas 5.0-U132p2 study from R2: platform, median cut-off modus). For all illustrated transcripts, the differences were significant, *p* < 0.05.

The clinical outcome data of patients with gliomas harboring low or high expressions of different fusogens and the tunneling membrane nanotube-related transcripts were obtained from Rembrandt, Madhavan-550 MAS.5.0-u133p2 study, R2: platform. The analyses of the overall survival of patients with gliomas harboring a low or high expression of the TNF Alpha Induced Protein 2 (TNFAIP2) transcript indicates that high expression levels of TNFAIP2 were associated with poor prognosis (*p* = 1.2 × 10^−4^). Moreover, upregulations of the TNFAIP2-stabilizing chaperone ERp29 [38], the TNFAIP2-associated small GTPase RALA, and S100A4 protein, which is responsible for the directional intercellular mitochondria transfer through the tunneling membrane nanotubes [39], significantly worsened patient outcome (*p* = 2.1 × 10^−9^, *p* = 3.3 × 10^−4^, and *p* = 7.4 × 10^−12^, respectively). High levels of ERVW-1, ERVK3-1, ERVK13-1, and CD9 fusogens were associated with overall poor survival as well (*p* = 1.5 × 10^−4^, *p* = 1.6 × 10^−3^, *p* = 0.04, and *p* = 2.3 × 10^−6^, respectively). Figure 2 illustrates the Kaplan–Meier overall survival curves for patients with gliomas harboring low or high expression levels of the above transcripts with a significant influence on overall patient survival.

Thus, our analysis confirms the enrichment of fusogens and the tunneling membrane nanotube-related transcripts in gliomas and their negative impact on overall patient survival.

## 4. HuR-Dependent Cell-Signaling Pathways of Cell Fusion and Tunneling Nanotube Formations Leading to Glioma Heterogeneity

**Figure 3:**
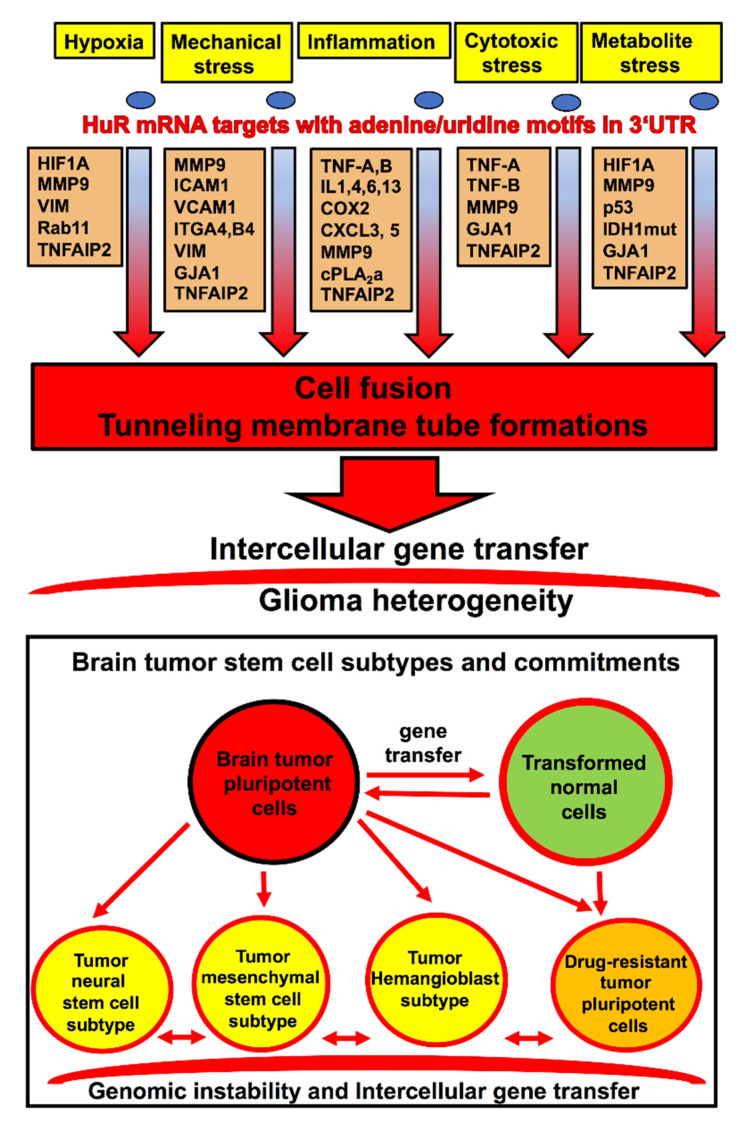
Schematic illustration of HuR-dependent stress response cell-signaling pathways involved in cell fusion and tunneling nanotube formations leading to glioma plasticity and heterogeneity.

Hypoxia, mechanical stress, chronic inflammation, cytotoxic stress, and oncometabolites associated with free radical formations are reported to potentiate intercellular membrane fusion events, and these conditions are often associated with the glioma microenvironment [14,20,21,22,23,27,35,44,45,46,47]. The mRNA-binding protein of ELAV-family HuR is a valuable biomarker of brain tumor progression [48,49,50,51] and is involved in the regulation of the key cell-signaling pathways responsible for the inflammatory glioma microenvironment, the hypoxia-related stress response, the transitions of classic and proneural glioma subtypes to the mesenchymal subtype, the metabolic stress, and the reactive oxygen species (ROS) generation associated with D-2HG oncometabolite production in low-grade gliomas harboring single alleles with IDH1-R132H/C/S mutations [52,53,54,55]. HuR exhibits strong overexpression in gliomas and shuttles from the nucleus to the cytoplasm to stabilize and promote the transfer and translation of mRNA transcripts enriched with adenine/uridine motifs in 3‘UTR [53,54,55,56]. Figure 3 summarizes the key transcripts, which positively correlate with the formation of tunneling membrane nanotubes and cell-to-cell fusion and are reported as being directly upregulated by HuR in gliomas.

The proinflammatory cytokines, including IL-1, IL-6, and TNF-alpha, increase the probabilities of lipid protrusions, essential for the cell-to-cell fusion, through the regulation of arachidonic acid (AA) and sphingolipid metabolisms [57,58]. Additionally, the cytosolic phospholipase A2-alpha (cPLA_2_α), which is known as the key enzyme that catalyzes the membrane glycerophospholipids at the sn-2 position to form AA, is the direct HuR-mRNA target upregulated in gliomas [58,59,60,61,62]. COX-2 (an inducible form of the cyclooxygenase enzyme that catalyzes the first step in the synthesis of prostanoids) is overexpressed in gliomas and is an established HuR-mRNA target. COX-2, in combination with PGE2, may influence ROS generation and controls the cellular redox state; therefore, it impacts cell fusion and tunneling membrane nanotube formation [63,64]. Direct COX-2 mRNA stabilization by HuR leading to an increase in COX-2 expression has been demonstrated in breast carcinoma [65]; also, the constitutive overexpression of COX-2 in ovarian and colon cancers is the result of HuR overexpression [63,66]. The positive interplay between HuR and COX-2 has been reported in gliomas as well [67].

Based on the combination of histological, ultrastructural, and genetic evidences, several models have emerged and described fusion steps, the diversity of chemokines, and a variety of cytoskeleton–lipid interactions leading to the formations of cell-to-cell extended contact zones, invasive protrusions, and tunneling membrane nanotubes [10,68]. However, all models imply the rearrangement of the actin-dependent cytoskeleton to match fusion machineries in the fusing cells. In most cases in mammalian cells, the cytoskeleton–lipid interactions during membrane fusion culminate with the road-like structures filled with bundles of parallel F-actin filaments, which allow the intercellular material transfer. Several lines of evidence suggest that the formation of these cytoskeletal intercellular roads is cytochalasin B-sensitive and employs several HuR-dependent chemokines, matrix metallopeptidase, GTPases, and cytoskeletal components (to include CXCL1, CXCL3, CXCL5, MMP9, ACTB, VIM, Ral11, RalA, RalBP1, Exo84, Sec5) [31,34,36,69,70,71,72,73,74]. It will be worth mentioning that some forms of membrane fusion (such as in the hypoxia condition) require the autophagy-dependent degradation of macromolecular membrane complexes. HuR promotes autophagosome formation by regulating the expression of several essential autophagy-related gene products (to include ATG5, ATG7, ATG12, ATG16L1) [75,76]. Hence, we conclude that the mRNA-binding protein HuR may serve as a potential positive regulator of the intercellular membrane fusions on the molecular level in gliomas.

Glioma-to-stromal cell communication via intercellular gene transfer leads to gene reprogramming in fused cells and the creation of de novo tumorigenicity and plasticity of glioma stem cells. The following glioma/microenvironmental transfers of biological materials via permanent and temporal cell fusions have been identified: (i) the bidirectional acquisition of the whole genome between glioma stem cells and bone marrow mesenchymal cells, between glioma stem cells and endothelial cells or pericytes, between glioma stem cells and monocytes (macrophages and microglia), between glioma cells and neural stem cells, and between glioma stem cells and macrophages fused with T cells [28,29,77,78,79,80,81,82,83]; (ii) the mitochondria and the cargo vesicle transfers through the tunneling membrane nanotubes between glioma cells and reactive astrocytes, between glioma and neuronal cells, between endothelial cells and pericytes in the glioma microenvironment, between glioma cells and macrophages, between glioma cells by themselves [22,23,30,31,32,33,34,84]; (iii) viruses and viral genome transfers between glioma cells by themselves, and between T cells and macrophages fused with glioma cells [32,33,85]. It is worth mentioning that proinflammatory monocytes and macrophages infiltrate the glioma microenvironment in a HuR-dependent manner and positively contribute to cell fusion and tunneling nanotube formations, and therefore promote glioma plasticity, tissue heterogeneity, and angiogenesis [86,87]. Moreover, growth factor- and integrin-mediated HuR expression and nuclear/cytoplasmic translocation have been reported in different cell types, which commonly reside in the glioma microenvironment [88,89,90]. Hence, we suggest that HuR overexpression stimulates the proinflammatory cellular composition of the glioma microenvironment, which provides positive feedback for HuR overexpression and nuclear/cytoplasmic shuttling in glioma cells and is associated with cell fusion and tunneling nanotube formations, which favor the development of glioma heterogeneity and treatment resistance.

## 5. Potential Pharmacological Modulators of Cell Fusion

Currently, several classes of inhibitors of cell fusion are under development worldwide: (i) the entry inhibitors, also known as the antiretroviral drugs, which inhibit fusogenic protein function; (ii) the F-actin depolymerizing agents (such as cytochalasin B), which inhibit actin-dependent cytoskeletal rearrangements essential for formations of the tunneling membrane nanotubes and the intercellular transfer; (iii) the modulators of the membrane fluidity; (iv) the inhibitors of autophagy; (v) the inhibitors of epithelial–mesenchymal and proneural–mesenchymal transitions in gliomas. The FDA-approved group of the entry inhibitors related to antiviral drugs includes 46 compounds, which represent the fusion-inhibitors, the CCR5 antagonists, and the post-attachment inhibitors. Although the actin-modifying agents exhibited strong potency in the inhibition of cell fusion in vitro, the actin-polymerization or depolymerization inhibitors are not the viable therapeutics due to their overall cytotoxicity. There are only two FDA-approved cytoskeletal modulators: (i) HALAVEN (eribulin mesylate), which has a broad spectrum of antitumor effects and is mostly utilized for the treatment of inoperable liposarcoma and metastatic breast cancer [91]; (ii) methyl-β-cyclodextrin (MCD) and its derivatives, which, as it was shown recently, induce actin depolymerization [92]. Interferon, Resveratrol, Miltefosine, Perifosine, Filipin, MCD, Emodin, monounsaturated and polyunsaturated fatty acids (MUFAs and PUFAs, respectively), diets with linoleic acid, oleic acid, marine fish oils are believed to be the perturbators of the cell membrane fluidity and are utilized for cancer treatment or prevention [93]. Chloroquine or hydroxychloroquine (HCQ) is an FDA-approved inhibitor of autophagy; ROC-325, Lys05, and DQ661 are the newest most potent inhibitors of autophagy from preclinical trials [94,95]. Note that HALAVEN, the actin-modifying agent, prevents glioma transitions to the mesenchymal subtype induced by the hypoxic condition. Pirfenidone (an inhibitor of the TGFb-activated pathways), Quetiapine (an inhibitor of the NF-kB cell-signaling pathways), Rifampin (an inhibitor of the Wnt/beta-catenin cell-signaling pathway), Naproxen (an inhibitor of the non-canonical Wnt signaling pathways), Itraconazole (an inhibitor of the Hh cell-signaling pathways), Metformin (an inhibitor of the mitochondrial respiratory chain complex one and an activator of AMPK kinase) are among the FDA-approved potential inhibitors of transition to the mesenchymal subtype [96]. Most of the above drugs are domain-, gene-, or pathway-specific and have to be utilized in combination with other drugs.

Cell fusion and tunneling nanotube formations are multistep processes, which require diverse sets of genes at different stages; therefore, many inhibitors of cell fusion are stage-dependent. For example, in the PC12 cells, cytochalasin B (the actin depolymerization drug) strongly interfered with the formation of the tunneling nanotubes, but hardly affected their numbers and stability after formation [31]. Wiskott–Aldrich syndrome protein (WASP)-deficient macrophages were able to resemble tunneling nanotubes structures; however, the material transfer in these structures was completely abolished [97]. Small GTPases, such as Ras, Cdc42, Rac1, and RhoA, have been reported to be involved in actin remodeling and tunneling nanotube formations in different cell types. The gene-specific knockdown or inhibition of the individual GTP-binding transcripts usually led to a decrease in tunneling nanotube formation by 20–40%, and rarely by 75–90% compared to the control [36,37,97,98,99], suggesting that multiple gene-sets might backup mechanisms of tunneling tube formation in the same cell. The steps of nanotube biogenesis, consisting of the tube formation, elongation, and degradation/disassembly, are affected differently, sometimes in opposite directions, by the same gene-sets, adding additional complexity to the development of the nanotube modulators [97]. In this regard, HuR inhibitors could be valuable tools in the modulation of cell fusion and tunneling nanotube formations, illuminating a way to suppress (i) the inflammatory and hypoxic microenvironment (the leading cause of the cell fusion in the gliomas and different types of cancer), (ii) the expression of the multiple sets of the fusogenic proteins and the tunneling nanotube-related transcripts, (iii) the transcripts essential for cytoskeleton remodeling and membrane protrusion formations, (iv) the transcripts and cell-signaling pathways involved in autophagy formation [100,101,102,103,104,105,106,107,108,109,110,111].

We propose that the HuR inhibitors may produce interference with cell fusion and tunneling membrane nanotube formations due to the fact that HuR is the central node in the regulation of inflammation, stress response, lipid metabolism, autophagy formation, and the actin-related cytoskeletal transcripts essential for cell fusion. To date, the following key compounds/scaffolds have been discovered as the inhibitors of HuR function: MS-444 as a blocker of HuR dimerization and nuclear/cytoplasmic shuttling [100]; CMLD1, CMLD2, quercetin, azaphilone derivatives, DHTS, NSC#84126, and mitoxantrone as the modulators of the HuR/mRNA interaction [101,102,103,104,105,106,107]; pyrvinium pamoate, okicenone, trichostatin, 5-aza-2′-deoxycytidine (AZA) as the inhibitors of HuR shuttling [100,108]. The pyrvinium pamoate, an antihelminthic drug, is FDA-approved and has been recently repurposed for the blocking of HuR nuclear/cytoplasmic shuttling [109]. Mitoxantrone is classified as an antitumor antibiotic and as an inhibitor of HuR/Cox2-mRNA interaction. Novantrone (mitoxantrone hydrochloride) injections for reducing neurologic disability and/or the frequency of clinical relapses in patients with secondary (chronic) progressive, progressive relapsing, or worsening relapsing–remitting multiple sclerosis have been approved by the FDA since October 2000 [110]. Several randomized multicenter trials have been initiated with Novantrone as anti-inflammatory drug for treatment of prostate, ovarian, breast, hematologic cancers, and solid or central nervous system (CNS) tumors overexpressing EGFR.

Inflammation has been recognized as one of the risk factors for oncogenic transformations in cancer [112,113]. Inflammatory mediators, such as prostaglandins (PGs), thromboxanes, and leukotrienes, are implicated in the inflammatory processes in the tumor microenvironment; PG production is tightly regulated by the COX-2 enzyme and favors tunneling tube formations. COX-2 selective non-steroid anti-inflammatory drugs (NSAIDs) and non-selective NSAIDs are considered as anti-inflammatory and antitumor chemotherapeutics [114]. Non-selective COX inhibitors, tolfenamic acid and indomethacin, substantially blocked both TNT formations and the spread of HCMV viruses between fibroblasts [115]. Recently, the anti-inflammatory and the TNT- and COX-1-inhibitory properties of NSAIDs have been confirmed by using (phendione)ZnII(NPR)_2_(H_2_O)_2_ and (phendione)ZnII(MFN)_2_ compounds on human breast cancer cell lines [116]. Although HuR inhibitors are currently available for preclinical evaluations, the combinations of the NSAIDs and gene-specific targeting approaches remain valuable as suppressors of cell fusion and tunneling nanotube formation.

**Figure 4:**
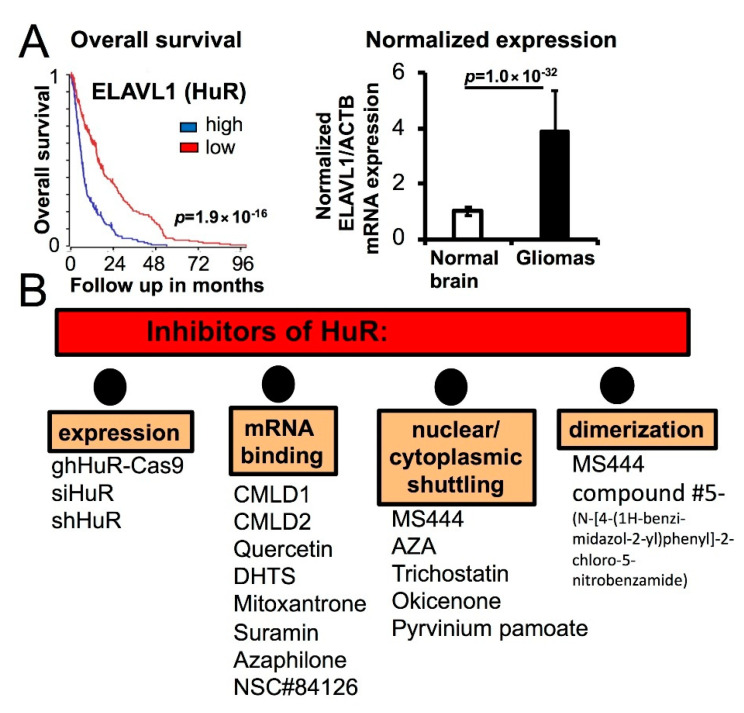
Classes of the available HuR inhibitors. (**A**) Graph (left) illustrates the Kaplan–Meier overall survival curves for patients with gliomas harboring low (red, *n* = 245) or high (blue, *n* = 245) expression of the ELAVL1 transcript (Rembrandt, Madhavan, Mas 5.0-U132p2 study from R2: platform, median cut-off modus); the graph (right) illustrates normalized ELAVL1/ACTB expression in gliomas (*n* = 500) compared to normal brain (*n* = 44). Data are shown as mean ± SD. The following research studies from the R2: platform have been utilized for the analysis: Rembrandt, Madhavan, Mas 5.0-U132p2 study for gliomas and Harris, Mas 5.0-U132p2 study for normal brain. (**B**). Classes of the available HuR inhibitors.

In our recent work, we developed a strategy to search and optimize the inhibitors of HuR dimerization in glioma cells [54]. HuR dimerization is essential for HuR nuclear/cytoplasmic translocation and the high-affinity binding to target mRNA [100]. HuR dimerization/multimerization is mostly observed in cancer cells and is associated with glioma progression [54]. The combination of medicinal chemistry with high-throughput HuR-specific biochemical and cell-based assays led us to the identification of several new inhibitors of HuR dimerization, such as compound #5 (*N*-[4-(1*H*-benzi-midazol-2-yl)phenyl]-2-chloro-5-nitrobenzamide), suitable for future optimization [54]. Figure 4 summarizes the impact of HuR overexpression on patient overall survival and illustrates classes of the available HuR inhibitors.

Several reports suggest that HuR may be directly involved in the intercellular membrane fusion during development and under the pathological conditions: (i) HuR-deficient mice are embryonic-lethal due to defects in placenta development [117]; (ii) HuR is essential for the skeletal muscle myotube formations during embryogenesis [118,119]; (iii) HuR contributes to the post-natal pathological angiogenesis via the regulation of pruning of the vascular branches and the endothelial cell self-fusion during this process [88,120,121,122]; on the other hand, HuR dimerization may promote atherosclerosis and may enhance the permeability of the vascular endothelial layer [123]; (iv) HuR is essential for the formation of germ cell syncytium where cells stay connected to one another by intercellular bridges [124]; (v) under the hypoxic condition, HuR enhances epithelial-to-mesenchymal transition, which is associated with intra- and intercellular microtubule formations and could be suppressed by inhibitors of HuR nuclear/cytoplasmic shuttling [125,126]; (vi) HuR promotes integrity of the gap-junction and the stability of Cx43 transcripts, which are involved in the type ii intercellular tunneling nanotube and microtube formations [127,128,129]. Collectively, HuR protein dimerization and translocation from the nucleus to the cytoplasm is associated with cell reprogramming toward cell differentiation and intercellular membrane fusion on several occasions during normal development. In the post-embryonic period, HuR is predominantly nuclear, is observed in spliceosomes, and may regulate transcriptome splicing to keep cell identity [130]. HuR is dimerized and shuttles to the cytoplasm under pathological conditions such as obesity, diabetes, cancer, and in response to cellular stress to promote cell motility, survival, plasticity, aberrant proliferation, and angiogenesis. Therefore, we suggest that glioma stem cells with cell-cycle checkpoint abnormalities, genomic instabilities, and renewal abilities hijack HuR function in stressed glioma microenvironments to advance glioma heterogeneity and proliferation. Hence, we predict that the inhibitors of the HuR nuclear/cytoplasmic shuttling and dimerization could be valuable pharmacological tools for the inhibition of glioma cell plasticity and proliferation, and therefore the suppression of glioma progression at different stages of disease development.

## 6. Conclusions and Perspectives

The elimination of glioma heterogeneity evoked by pharmacological/radio treatments or by environmental stress is an established chemotherapeutic goal. Understanding the role of HuR in the regulation of glioma heterogeneity in the tumor microenvironment is a new direction, which may lead to the discovery of common and environment-specific mechanisms of glioma plasticity. The implication of the HuR protein inhibitors in the treatment of glioma heterogeneity is new and has the following rationales: (i) HuR is overexpressed in gliomas; (ii) HuR orchestrates stress responses and chemoresistance; (iii) HuR upregulates cell-signaling pathways and mRNA transcripts essential for the intercellular membrane fusions and the tunneling membrane tube formations leading to the glioma cell survival and plasticity.

## Figures and Tables

**Table 1:** Expression of the fusogens and the fusogen receptor transcripts in PDGx neurospheres of different subtypes: proneural (PDGx1, PDGx2), classic (PDGx3, PDGx4), and mesenchymal (PDGx5).

**Endogenous Retroviral Fusogens**					
	PDGx1	PDGx2	PDGx3	PDGx4	PDGx5
ERVK13-1	+++	+++	+++	+++	+++
ERV3-1	++	+++	+++	+++	++
ERVMER34-1	+	+	+	+	+
ERVW-1	+	+	+	+	none
EBI3	none	none	+	none	++
ERVV-1	none	none	+	none	none
ERVV-2	+	none	none	+	none
**Fusogen Receptors**					
	PDGx1	PDGx2	PDGx3	PDGx4	PDGx5
CTNNB1	+++	+++	+++	+++	+++
ICAM1	none	+	+	+	+++
ITGA4	none	++	+	++	++
ITGB4	+	+	+	++	++
VCAM1	none	none	++	none	+
**Germ Cell Fusogens**					
	PDGx1	PDGx2	PDGx3	PDGx4	PDGx5
CD9	+++	+++	+++	+++	++
IZUMO1	+	+	+	+	none
IZUMOR1	+	+	+	+	none
IZUMO3	none	+	none	none	none
IZUMO4	++	++	++	++	+
SPA17	++	++	++	++	+
SPAG8	+	+	+	+	+
**Myoblast Fusion Factors**					
	PDGx1	PDGx2	PDGx3	PDGx4	PDGx5
MYMK	none	none	none	none	+
MYMX	+	none	+	none	+

**Table 2:** Expression of the tunneling nanotube-related transcripts in PDGx neurospheres of different subtypes: proneural (PDGx1, PDGx2), classic (PDGx3, PDGx4), and mesenchymal (PDGx5).

TNT Type i and Type ii Transcripts					
	PDGx1	PDGx2	PDGx3	PDGx4	PDGx5
TNFAIP2	+++	++	++	+++	+++
RALA	+++	+++	+++	+++	++
EXOC6	++	++	+++	+++	++
S100A4	+++	+	+++	++	+++
GJA1	+++	+++	+++	+++	none
GAP43	+++	+++	+++	+++	+

## Abbreviations

AA	Arachidonic Acid
CNS	Central Nervous System
CARs	T cells expressing Chimeric Antigen Receptors
cPLA_2_α	cytosolic Phospholipase A2-alpha
COX2	Cytochrome Oxidase Subunit 2
FDA	Food and Drug Administration
GEO	Gene Expression Omnibus repository database
HCQ	Hydroxychloroquine
MCD	Methyl-β-cyclodextrin
Novantrone	mitoxantrone hydrochloride
NSAIDs	Non-Steroid Anti-Inflammatory Drugs
Optune treatment	an application of the electric field, which induces apoptosis in rapidly dividing tumor cells
PDGx	Patient-Derived Glioma Xenografts
PGE2	Prostaglandin E_2_
PG	Prostaglandins
ROSc	Reactive Oxygen Species
R2:	Genomic Analysis and Visualization platform R:
TMZ	Temozolomide
TNFAIP2	TNF Alpha Induced Protein 2
TNT	Tunneling Membrane Nanotubes
MT	Membrane Microtubes
WASP	Wiskott-Aldrich Syndrome Protein
